# A new concept for cancer therapy: out-competing the aggressor

**DOI:** 10.1186/1475-2867-8-19

**Published:** 2008-12-12

**Authors:** Thomas S Deisboeck, Zhihui Wang

**Affiliations:** 1 Complex Biosystems Modeling Laboratory, Harvard-MIT (HST) Athinoula A. Martinos Center for Biomedical Imaging, Massachusetts General Hospital, Charlestown, MA, 02129, USA

## Abstract

Cancer expansion depends on host organ conditions that permit growth. Since such microenvironmental nourishment is *limited *we argue here that an autologous, therapeutically engineered and faster metabolizing cell strain could potentially *out-compete *native cancer cell populations for available resources which in turn should contain further cancer growth. This hypothesis aims on turning cancer progression, and its microenvironmental dependency, into a therapeutic opportunity. To illustrate our concept, we developed a three-dimensional computational model that allowed us to investigate the growth dynamics of native tumor cells mixed with genetically engineered cells that exhibit a higher proliferation rate. The simulation results confirm *in silico *efficacy of such therapeutic cells to combating cancer cells on site in that they can indeed control tumor growth once their proliferation rate exceeds a certain level. While intriguing from a theoretical perspective, this bold, innovative *ecology*-driven concept bears some significant challenges that warrant critical discussion in the community for further refinement.

## Background and hypothesis

**Figure 1:**
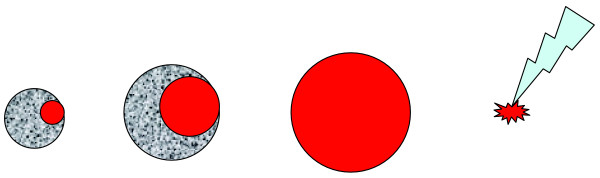
The time series schematic depicts the growth of the *red*, therapeutically engineered (tumor) cell clone within a native (*grey*) tumor cell population (patterning represents inhomogeneity of the cell population). Once the altered clone dominates the tumor population on site, it could be selectively therapeutically targeted.

Amongst the distinct hallmarks of cancer are uncontrolled growth and extensive cellular heterogeneity [1]. The 'ecology' concept here is based on the analogy that the host organ serves as 'bio-habitat' for a rapidly expanding heterogeneous tumor cell population, and that the organ's distinct microenvironmental conditions on site only support a certain tumor growth rate and overall tumor mass – prior to the onset of metastasis [2]. If so, one wonders if a tumor could be 'out-competed' for habitat dominance by an autologous cell population that has been engineered to outgrow the tumor cell populations, yet – other than the native cancer cells – can be therapeutically controlled. One can imagine a primary, autografted tumor cell line established from the patient's own tumor (biopsied at the time of operation) that has been genetically engineered to carry an on-off switch that can trigger programmed cell death, or apoptosis, 'on demand'. The corner stone of this innovative concept is to therapeutically skip any number of tumor progression steps by deliberately inserting an autologous cell population that securely *outperforms *even the most aggressive native cancer cell clone (see Figure 1).

The *performance *requirements for this therapeutic cell population include **(i) **its proliferation rate must exceed that of the most aggressive native tumor cells; **(ii) **it should exhibit a reduced apoptotic rate, and, **(iii) **it must exhibit a high metabolic consumption rate – thereby quickly exhausting the limited resources available to the native tumor cells on site. Assuming that tumor growth is bound by the microenvironmental conditions, we have defined already in [2] the quantitative relationship between the tumor growth rate and an organ's carrying capacity, *C*_*C *_as:

(1) $$\frac{\Delta V_{Tum}}{\Delta t}\leq \frac{\Delta C_{C}}{\Delta t}$$

Understanding that *V*_*Tum *_represents the composite volume of multiple native clones, with distinct proliferative phenotype, and arguing that the therapeutic cell population, *P*_*T*_, must outgrow the native tumor, we revise **Eq. (1) **to:

(2) $$\left(\frac{\Delta V_{P_{T}}}{\Delta t}>>\sum \left(\frac{\Delta V_{Clone_{1}}}{\Delta t}+\frac{\Delta V_{Clone_{2}}}{\Delta t}+...\frac{\Delta V_{Clone_{n}}}{\Delta t}\right)\right)\leq \frac{\Delta C_{C}}{\Delta t}$$

From **Eq. (2) **follows first that our competition concept should hold primarily at tumor growth stages *prior *to reaching *C*_*C*_, ahead of the onset of metastasis. However, within that limit one can argue for some flexibility, precisely due to the impact of the tumor. That is, as detailed in [2], *C*_*C *_is defined as the ratio of an organ's composite volume infrastructure and the physiological functionality it has to provide. Both, a tumor-induced improvement in growth permission or nourishment (e.g., through angiogenesis or cooperative paracrine secretion of growth factors) as well as any cancer growth related (e.g., proteolytic) reduction of tissue functionality would yield an increase in *C*_*C*_. The following section describes the *in silico *model developed to test our hypothesis on inducing 'therapeutic competition'.

## Methods

To investigate the effects of engineered cells on the growth of native tumor cells, we present here a three-dimensional (3D) agent-based model that simulates the growth dynamics of both types of cells in parallel. An agent-based model can exhibit aggregated complex behavior patterns upon interactions among agents, and between agents and their environments [3]. Specifically, in modeling cancer systems, an agent often represents an individual cell [4-8]. For now, native tumor cells and engineered cells have the same metabolic and apoptotic rates (an assumption that will be relaxed in future works), but have distinctively *different *proliferation rates. In our model here, each cell is capable of gaining a certain number of 'proliferation' credits (PCs) at every point in time. If accumulated PCs in a given cell exceed a set threshold, the cell is eligible to proceed with proliferation. This proliferation threshold is pre-defined and (for now, reflecting ubiquitous metabolic house keeping in the same cell lineage) equal for both cell types (an assumption that, again, can easily be relaxed later on). In our model, this proliferation threshold is currently set to 100; however, when a cell has collected (at least) 100 PCs, it does not necessarily mean that the cell will immediately start proliferating; rather, it will have to meet some other microenvironmental conditions (see below). Native tumor cells and engineered cells gain distinct amounts of PCs at every time step, reflecting different proliferative capabilities (rates) of the two different cell types. We denote PC_TC _for the PCs for a native tumor cell, and PC_EC _for an engineered cell. In accordance with our concept (see also **Eq. (2)**), PC_EC _should always be higher than PC_TC_. Finally, we purposely set the range of PC_TC _to 1~33 such that we will have enough bandwidth left (i.e., 34~99; the maximum proliferation rate going to be tested is 99, because the threshold for proliferation has been set to 100) for PC_EC _in examining the dynamics of how engineered cells combat native tumor cells.

### Tumor growth environment

**Figure 2:**
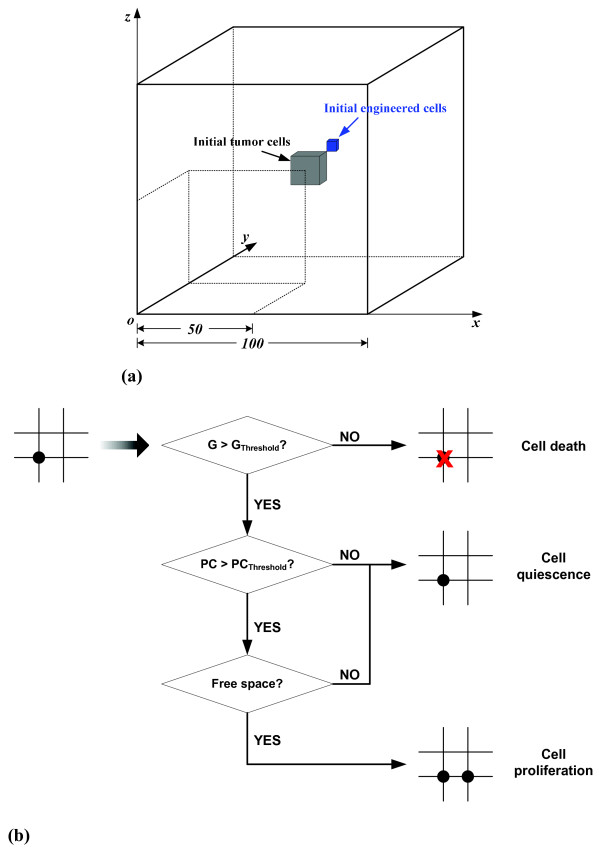
**(a) **Three-dimensional tumor growth environment with 100 × 100 × 100 grid points. Native tumor cells and engineered cells are randomly distributed in different cubes, indicated by *gray *and *red*, respectively. **(b) **Cell phenotypic decision algorithm. *G *represents glucose and *PC *represents proliferation credit; *G*_*Threshold *_and *PC*_*Threshold *_denote the threshold for glucose and proliferation rate, respectively.

The tumor's 3D virtual microenvironment is represented by a discrete cube of 100 × 100 × 100 grid points. Initially, 50 native tumor cells are randomly seeded in a smaller cube (5 × 5 × 5 grid points) that is located at the center of the larger, microenvironmental cube. The initial number of engineered cells implanted is 10, randomly distributed in a cube consisting of 3 × 3 × 3 grid points. The position of this 'therapeutic' cube can be any of the 8 vertices of the cube for native tumor cells. A certain amount of nourishment, represented by glucose, is uniformly distributed in the 3D environment and will be consumed by each viable cell. Figure 2(a) summarizes the setup of the 3D environment.

### Cellular phenotype

Three cell phenotypes are currently considered: proliferation, quiescence, and apoptosis or death, each for both cell types. Figure 2(b) schematically illustrates our algorithm on determining phenotypic changes. In brief, a cell will die if its nourishing, on-site glucose concentration drops below a pre-defined threshold. The threshold value and glucose consumption rate of a live cell are obtained from an *in vitro *study on mammary carcinoma cell spheroid growth [9], and have been rescaled to fit our model (for more details, please see our previous works [7,10]). A cell starts to proliferate if 1) its PCs exceed the proliferation threshold, as described above, and if 2) the onsite glucose concentration is sufficient, i.e. meets the requirement of keeping a cell alive. The cell then starts to search for an appropriate location for its offspring to reside in (candidate locations are the six grid points surrounding the cell). In our model, the most appropriate location is the one with the highest glucose concentration; if there is more than one location meeting this condition, the cell will randomly choose one. When a cell cannot find an empty location (i.e., a vacant grid point) to proliferate into, it will remain quiescent and continue to search for an empty location at the next time step. For simplicity, all initial native tumor cells and engineered cells start with a quiescent state.

### Tumor growth law

Tumor growth has been described by using a range of kinetics including for instance the Gompertz law and, more recently, the so called 'universal' law [11-14] which is based on underlying metabolic concepts and thus used in here. That is, within the range of 1~33, we attempt to test and find the most appropriate PC_TC_, i.e., the PC_TC _which best fits the universal tumor growth law. As reported in [11], the relative amount of energy devoted to tumor growth can be related to the proportion Δ*N/N *of the cells contributing to the growth,

(3) $$\frac{\Delta N}{N}\approx e^{-\tau }$$

where *N *is the total tumor cell number and Δ*N *is the difference between the rates of generated to dead tumor cells at one time step; *τ *represents the rescaled dimensionless time and can be calculated by using the following equation [12]:

(4) $$\tau =0.25aM^{-0.25}t-\ln(1-{\left(\frac{m_{0}}{M}\right)}^{0.25})$$

where *m*_0 _is the tumor mass at origin (t = 0), *M *is the final mass, and *a *is a parameter relating to a tumor's characteristics, such as its ability to metastasize or invade. According to the model's setup, a total of 50 native tumor cells are initially placed in the center of the cube. A simulation run elapses for 100 time steps, and each simulation run generates a time-series data of (Δ*N/N*). As a result, there will be a total of 33 sets of such time-series data being generated, corresponding to 33 possible PC_TC_.

We selected three sets of tumor growth parameters (i.e., with different *a*, *m*_0_, and *M *in **Eq. (4)**; see Table 1), representing *in vitro*, *in vivo*, and clinical tumor data, respectively. Three sets of time-series data of *e*^*τ *^will then be generated (see **Eq. (4)**). To seek the most appropriate PC_TC _for each of the three sets of *e*^*τ *^obtained using the universal law, we calculate correlation coefficients between **(1) **the 33 sets of time-series data from our simulation results (Δ*N/N*), and **(2) **the time-series data of *e*^*τ*^. This analytical process results in a number of 33 correlation coefficients for each of the three time-series data (i.e., *e*^*τ *^for *in vitro*, *in vivo*, and clinical tumor data, respectively). Statistically, a correlation coefficient is used for measuring the degree of closeness of two variables; hence, the correlation coefficient here is a measure of how well the simulated tumor growth data (Δ*N/N*) fit with the three types of real biomedical data: the higher the correlation coefficient, the stronger is the (positive) relationship between the two data sets compared.

**Table 1:** Parameters for selected tumors

	**Tumor**	**m_0 _(g)**	**M (g)**	**a (g^0.25^/day)**	**Reference**
*in vitro*	Human Glioblastoma	0.025	3	0.075	[25]
*in vivo*	Murine adenocarcinoma	0.2	8	0.37	[26]
*clinical*	Human breast cancer	1	646	0.81	[27]

## Results

The model was developed in C/C++ and is based in part on our previously presented agent-based modeling platforms [10,15].

**Figure 3:**
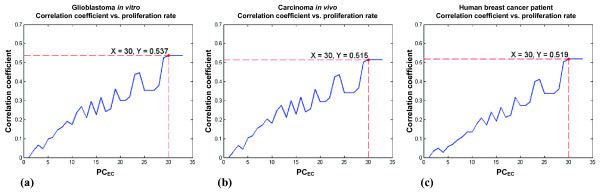
Correlation coefficient (*y-axis*) plots of the tumor growth data obtained from the universal law and simulations varying the value of PC_TC _(*x-axis*). **(a) **Human Glioblastoma *in vitro *data; **(b) **mouse adenocarcinoma *in vivo *data. **(c) **Human breast cancer data from patients.

**Figure 4:**
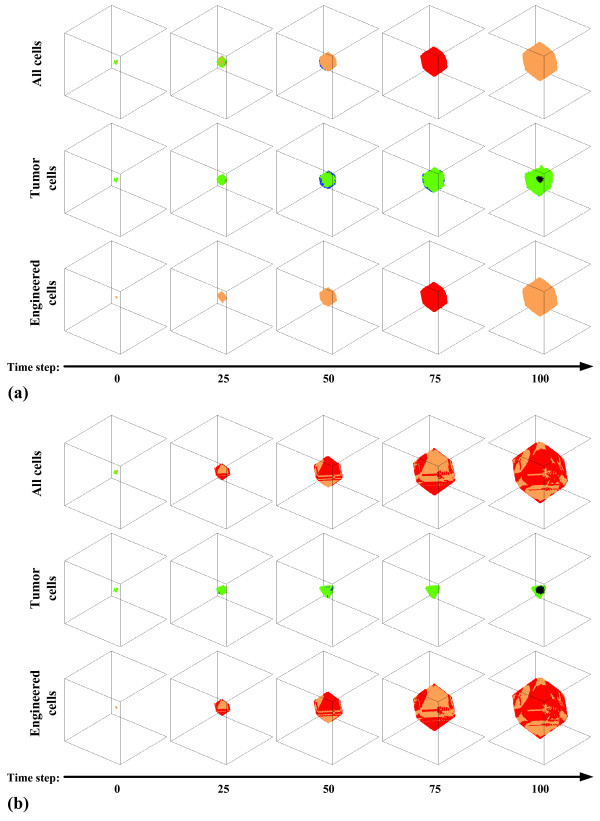
Growth of native tumor and engineered cells for **(a) **PC_TC _= 30 and PC_EC _= 34, and **(b) **PC_TC _= 30 and PC_EC _= 99. In both **(a) **and **(b)**, from *top *to *bottom*, results are displayed for all cells, native tumor cells and engineered cells, respectively. Note: proliferative native tumor cells are labeled in *blue*, quiescent native tumor cells in *green*, proliferative engineered in *red*, quiescent engineered cells in *orange*, and dead cells for both cell types are labeled in *grey*.

First, the most appropriate proliferation rate for tumor cells, PC_TC_, was determined using the model. Figure 3 shows correlation coefficients between the tumor growth data generated by our simulations and by the universal law, for **(a) ***in vitro*, **(b) ***in vivo*, and **(c) **clinical data, respectively. In all three panels, the correlation coefficient values peaked at numbers: 30~33. This means that when PC_TC _lays within 30~33, i.e., when a tumor cell receives 30~33 PCs at each time point, the simulated tumor growth follows the universal law the closest; hence, we chose to set PC_TC _= 30 for the subsequent simulations in which both types of cells were taken into account. We performed simulations, all with PC_TC _= 30, to investigate effects of different values of PC_EC _within a range of 34~99 on suppressing the growth of tumor cells. The maximum simulation step was again set to 100. Figure 4 displays two representative simulation results, with **(a) **PC_EC _= 34 and **(b) **PC_EC _= 99. The final cell populations of **(b) **exceed that of **(a) **due to a larger number of newly generated ECs.

**Figure 5:**
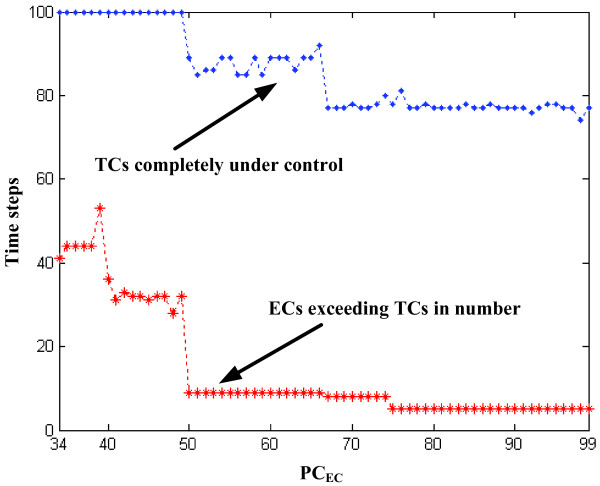
Effects of change in PC_EC _(*x-axis*) on out-competing native tumor cells (TCs). For each PC_EC_, two critical points are depicted: first, when engineered cells (ECs) exceed TCs in number (dashed line in *red *with star markers) and then when all TCs are surrounded by ECs (dashed line in *blue *with point markers), resulting in tumor growth inhibition.

However, comparing the distinct cell populations only is insufficient for determining 'success' of our concept; that is, at any step, even if the number of engineered cells by far outweighs the number of native tumor cells, conceivably some cancer cells continue to proliferate, hence may be able to escape. Therefore, we sought two critical time points in investigating the growth dynamics of the mixed cell population. The first is the time at which the number of engineered cells starts to exceed the number of native tumor cells, whereas the 2nd critical point is reached when native tumor cells seize to grow which in turn indicates that engineered cells established control. The effects of different PC_EC _on ECs out-competing TCs are shown in Figure 5, where for each PC_EC_, the corresponding two critical points are depicted. Overall, expectedly, ECs exceed TCs in number *prior *to controlling them. The plot also shows that, a higher EC proliferation rate (i.e., higher PC_EC_) leads to reaching the 2nd critical time point faster than a lower PC_EC _does. However, while a PC_EC _of 67 leads to the fastest control, any further increases fail to show added therapeutic value.

**Figure 6:**
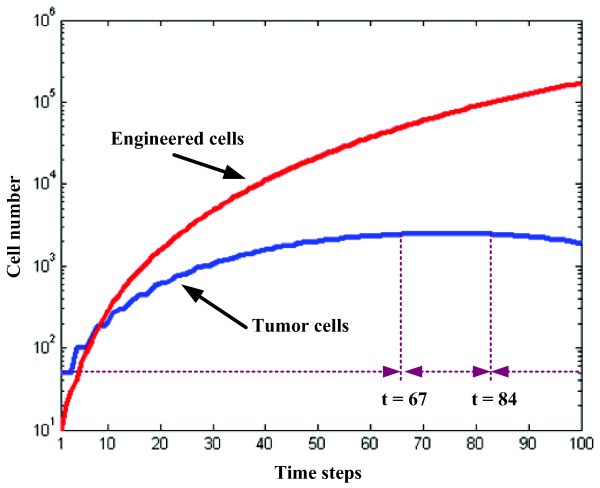
Cell number (logarithmic scale; *y-axis*) vs. time step (linear scale; *x-axis*) for a simulation with PC_TC _= 30 and PC_EC _= 67 (see Figure 5); native tumor cells are shown in *blue*, engineered cells in *red*.

Finally, the growth dynamics of the mixed cells with PC_TC _= 30 and PC_EC _= 67 are shown in Figure 6. As the simulation progresses, the number of ECs continuously increases, whereas the growth of TCs shows an interesting pattern in that up until time step 76, the number of TCs continuously increases, while it begins to decline thereafter. This result demonstrates that native tumor cells, once completely controlled, seem to be unable to regrow. To investigate these patterns further, we divided the cancer growth into three phases: TCs will first experience a phase (t = 1–66) where they continue to grow while they start *competing *with ECs for limited resources. Growth then enters a phase (t = 67–83) where the number of alive TCs remains *stable *in that the number of newly generated TCs is equal to those that become apoptotic. Finally, the cancer volume starts to *decrease *(t = 84–100) as the rate of TC growth becomes negative.

## Discussion and conclusions

Despite undeniable progress over the last decades, overall the clinical outcome of many common cancer types remains discouraging [16]. For 2008, in the Unites States alone, a total of 1,437,180 new cancer cases and 565,650 deaths from cancer are estimated [17]. As such, new, bold concepts are desperately needed. Here, we propose that, based on the reasonable argument that cells depend in their metabolism on limited microenvironmental permission, one could potentially attempt to deplete and ultimately outgrow a solid tumor by deliberately introducing a population of rapidly metabolizing, *therapeutic *cancer cells. (To reduce the threat of immunologic rejection) these autologous cells would be harvested via biopsy from the patient's own tumor, *ex vivo *genetically engineered to bolster their growth rate while inserting effective safe-guards, and then re-injected on site to eventually control tumor cells solely by competing more successfully for limited resources. We note that, originally, the now highly publicized paradigm of anti-angiogenesis was built on the very same premise, i.e. to therapeutically reduce vascularization and thus starve the tumor of the extrinsic nutrients it so critically depends on [18]. Our concept, however, is based on the introduction of *intrinsic *competition and to illustrate it, we have developed a computational, agent-based model where engineered cells differ from native tumor cells in their proliferation rate. We simulate the efficacy of such therapeutic cells, exhibiting different proliferation rates, on out-competing and eventually controlling native tumor cells.

The difference in the resulting growth patterns of tumors (sub-figures in the 2nd row of Figure 4(a) and 4(b)), with PC_EC _= 34 and PC_EC _= 99, may seem surprising at first since the PC_TC _for both simulations has been the same. However, this can be explained by the fact that the more engineered cells are generated, the more likely it is that an empty location in the cube is occupied by these cells; thus, engineered cells in a simulation with higher PC_EC _rapidly enlarge their domain, which in turn prevents native tumor cells from replication since the possibility of finding an empty lattice location for their offspring to reside in is becoming increasingly low. Furthermore, not only the resulting growth patterns but also the tumor volumes (i.e., number of viable tumor cells) are different. That is, the one in **(b) **is smaller than that in **(a)**, which implies that tumor cells are controlled more effectively in **(b)**. For instance, at time step 100, tumor cells are still proliferating in **(a)**, while in **(b) **they have already become entirely growth-suppressed. Thus, higher PC_EC _achieved faster tumor suppression which is the result of a sequence combining growth arrest (competition for space) with subsequent cell death (competition for nourishment) as illustrated in Figure 6. Based on our results, a two fold increase in EC proliferation rate (PC_EC_) led to a marked acceleration in tumor control; also, there is an *optimum *value for PC_EC_, that is, increasing PC_EC _beyond 67 fails to add therapeutic value (Figure 5). Together, this argues for a target range in engineering these cells to replicate faster. To provide more insights into a potential clinical scenario, we divide the observed TC dynamics into three phases (see Figure 6). While these time frames very likely depend on a number of parameters in addition to the proliferation rates, such as metabolic and apoptotic rates as well as organ type and thus specific carrying capacity, existence of this last phase – where tumor growth is not only controlled but tumor cells actually start to decline in number – seems to support our *in silico *concept and therefore warrants further investigations.

However, to provide more detailed quantitative insights into the relationship between tumor cells and engineered cells, we need to amend the current setup in future works. First, we will need to explore the impact of different metabolic rates; that is, engineered cells have to sustain a higher proliferation rate and thus their metabolic consumption should be distinct to reflect the demand; this may require a dynamic adjustment of the proliferation threshold. However, qualitatively, coupling of the engineered cells' higher proliferation rate to a more pronounced metabolic consumption rate, should only accelerate tumor control, hence is largely a means to contain growth of the therapeutic cell line itself. Second, the current microenvironment is overly simplified as only glucose plays a role in determining cell phenotypic transitions. As such, other key environmental factors, such as gradients in oxygen and growth factors [19-22], can be integrated into the lattice. Thirdly, heterogeneous cells should not only compete with each other for limited nutrient resources, but may also be able to co-exist and potentially even cooperate for performing physiological activities [2].

While the underlying *ecology *concept of therapeutically exploiting *controlled *progression to outperform and overgrow the native tumor cell population on site by an aggressively expanding yet therapeutically manageable clone is strikingly simple, careful consideration reveals a number of very significant technical challenges involved in putting this concept into practice. Those include **(i) **a potentially *inductive *effect for local tumor invasion and distant metastasis. This is based on the hypothesis that tumors tend to increase their overall surface through spatio-temporal expansion in an effort to avoid the limits imposed by diffusive yet dwindling microenvironmental supplies [23]. Since the metabolism of the engineered cells practically reduces the carrying capacity, *C*_*C*_, the incentive for tumor cells to start invasion and accelerate metastasis should be increased (see **Eq. (1)**). Tumor induced neovascularization should temporarily stabilize *C*_*C*_, and as such, simultaneous anti-angiogenetic therapy would likely increase the effectiveness of our proposed approach, while adding anti-invasive measures, as far as available, should increase its safety. Related critical issues are **(ii) **how can one ensure that the therapeutic clone remains genetically stable and thus phenotypically robust so that it neither risks being outdone by the tumor's own ability to progress under stress, nor that it itself mutates to an uncontrollably aggressive strain? Moreover, since the approach is based on the notion of competition amongst cells, any evolvement of *cooperation *[24] could jeopardize the therapeutic result. Lastly, **(iii) **which therapeutic safeguards have to be inserted where to guarantee precise monitoring of the engineered clone *in situ*, and how, when and where can these therapeutic cells be targeted safely and effectively to avoid that they *themselves *become a risk for the patient (by e.g. increasing biomechanical pressure on site)?

While this list of technical challenges is by no means exhaustive yet surely already daunting, the theoretical appeal of an innovative, ecology-concept driven therapy that turns the tumor's well known ability to progress under stress into a therapeutic virtue is undeniable, hence should warrant further *in silico *and experimental investigations into its potential risks and benefits.

## Competing interests

The authors declare that they have no competing interests.

## Authors' contributions

TSD conceived the idea and developed the concept, designed the model, drafted and revised the entire manuscript. ZW contributed to the development of the model as well as analysis and interpretation of the data. Both authors read and approved the final manuscript.
